# Intracellular proteolysis of kininogen by malaria parasites promotes release of active kinins

**DOI:** 10.1186/1475-2875-11-156

**Published:** 2012-05-07

**Authors:** Piero Bagnaresi, Nilana MT Barros, Diego M Assis, Pollyana MS Melo, Raphael G Fonseca, Maria A Juliano, João B Pesquero, Luiz Juliano, Philip J Rosenthal, Adriana K Carmona, Marcos L Gazarini

**Affiliations:** 1 Departamento de Biofísica, Universidade Federal de São Paulo, Rua Pedro de Toledo, 669 – 7 andar, 04039-032, São Paulo, Brazil; 2 Departamento de Ciências Exatas e da Terra, Universidade Federal de São Paulo, Rua Prof. Artur Riedel, 275, 09972-270, Diadema, São Paulo, Brazil; 3 Department of Medicine, San Francisco General Hospital, University of California, 1001 Potrero Avenue, San Francisco, California, 94110, USA; 4 Departamento de Biociências, Universidade Federal de São Paulo, Av. Ana Costa, 95, 11060-001, Santos, São Paulo, Brazil

## Abstract

**Background:**

The malaria burden remains a major public health concern, especially in sub-Saharan Africa. The complex biology of *Plasmodium*, the apicomplexan parasite responsible for this disease, challenges efforts to develop new strategies to control the disease. Proteolysis is a fundamental process in the metabolism of malaria parasites, but roles for proteases in generating vasoactive peptides have not previously been explored.

**Results:**

In the present work, it was demonstrated by mass spectrometry analysis that *Plasmodium* parasites (*Plasmodium chabaudi* and *Plasmodium falciparum*) internalize and process plasma kininogen, thereby releasing vasoactive kinins (Lys-BK, BK and des-Arg^9^-BK) that may mediate haemodynamic alterations during acute malaria. In addition, it was demonstrated that the *P. falciparum* cysteine proteases falcipain-2 and falcipain-3 generated kinins after incubation with human kininogen, suggesting that these enzymes have an important role in this process. The biologic activity of peptides released by *Plasmodium* parasites was observed by measuring ileum contraction and activation of kinin receptors (B1 and B2) in HUVEC cells; the peptides elicited an increase in intracellular calcium, measured by Fluo-3 AM fluorescence. This effect was suppressed by the specific receptor antagonists Des-Arg^9^[Leu^8^]-BK and HOE-140.

**Conclusions:**

In previously undescribed means of modulating host physiology, it was demonstrated that malaria parasites can generate active kinins by proteolysis of plasma kininogen.

## Background

Multiple proteolytic enzymes have been identified in *Plasmodium* species and appear to be involved in several important aspects of parasite biology [1,2]. Notably, cysteine and aspartic proteases are implicated in the hydrolysis of haemoglobin [3,4] to provide free amino acids for protein synthesis by intraerythrocytic parasites. Cysteine and aspartic protease inhibitors block this process [5] and kill parasites at nanomolar concentrations [6]. Recent studies demonstrated that the *Plasmodium falciparum* cysteine proteases falcipain-2 and falcipain-3 act with similar specificity in haemoglobin degradation, not via an ordered hydrolytic pathway, but through rapid hydrolysis at multiple sites [7]. Falcipain-2 knockout parasites showed reduced haemoglobin degradation while falcipain-3 knockouts were not viable, suggesting that late expression of falcipain-3 rescued falcipain-2 knockouts and that falcipain-3 is essential for intraerythrocytic development [8,9]. Considering the important involvement of proteases in *Plasmodium* biology, the roles of these enzymes in cellular and biochemical events are targets of active investigation.

The pathophysiology of malaria is poorly understood, but endothelial cell activation and adherence of infected erythrocytes to endothelial cells appear to be important features in pathogenesis [10,11]. The contact of adherent infected red blood cells with endothelium can induce vessel wall shear stress, stimulating the release of the potent vasodilator nitric oxide [12]. However, it is unclear if nitric oxide is beneficial or harmful in malaria [13]. In fact, when endothelium is disturbed, cells can exhibit a wide range of biochemical responses [6], including bradykinin (BK) release [14]. Kinins are biologically active peptides released from a multifunctional plasma protein, kininogen (HK). They can induce vasodilatation, stimulate the production of nitric oxide, activate endothelial cells, enhance microvascular permeability and modulate the metabolism of different tissues [15-19]. It has been reported that cysteine proteases from some pathogenic microorganisms, including *Trypanosoma cruzi*[20,21], *Porphyromonas gingivalis*[22] and *Fasciola hepatica*[23], release kinins. During the *Trypanosoma cruzi* infection, immature dendritic cells (DC) sense the presence of the parasite in the peripheral and lymphoid tissues by B_2_R stimulation mediated by bradykinin, which is generated by action of its major cysteine protease, cruzipain. These now activated DC trigger a cascade of immune cells activation, culminating in the generation of immunoprotection by IFN-**γ-**producing T cells [24]. The B_2_R activation also potentiates *Trypanosoma cruzi* invasion of host cells [25]. *Porphyromonas gingivalis* is also able to release kinin by its own proteolytic activity. These kinins promote B_2_R pathway activation that, in conjunction with TLR2 activation by the bacterial LPS, modulates effector T cells commitment in the pathology [26].

However, kinin formation in *Plasmodium* infection has been poorly explored. Older reports suggested that kinins may be involved in malaria pathology [27,28] and a reduction of plasma kininogen was observed in mice infected by *Plasmodium berghei*[29]. Nonetheless, the mechanisms by which kinins are generated and the biological effects of released kinins have not previously been reported. In the present study, it is shown that malaria parasites take up plasma proteins from the host and process these proteins to generate biologically active peptides.

## Methods

### Parasites

*Plasmodium chabaudi* (clone AS) was maintained in Balb/C mice by weekly transfers from previously infected mice. Animals infected at the trophozoite stage (parasitaemia ~60%) were sacrificed, blood was collected, and leukocytes and platelets were removed from whole blood by filtration through a powdered cellulose column (Whatman CF11). Trophozoite-infected erythrocytes were then washed three times by centrifugation at 1,500 g for 5 min in phosphate buffered saline (PBS; 137 mM NaCl, 2.7 mM KCl, 4.3 mM Na_2_HPO_3_, 1.4 mM Na_2_HPO_3_, pH 7.4). Parasites (10^7^ cells/ml) were isolated by lysis of erythrocyte membranes with 10 mg/ml saponin in PBS. After pelleting to remove red cell membranes, the parasites were washed twice in PBS by centrifugation at 2,000 g at 4°C.

*Plasmodium falciparum* (3D7 strain) was cultured in RPMI 1640 medium supplemented with 10% human serum as previously described [30] and parasites were isolated from erythrocytes when cultures reached ~5% parasitaemia using the same procedure employed for *P. chabaudi*.

### Kinin generation by *Plasmodium* parasites

Isolated parasites (*P. chabaudi* and *P. falciparum*) (10^4^ cells/ml) were incubated with 0.35 μM of human high molecular weight kininogen (HK) or with the synthetic fluorogenic peptide Abz-MISLMKRPPGFSPFRSSRI-NH2 (30 μM; Abz = *ortho*-aminobenzoic acid) for 5 min, 10 min, 30 min, 1 and 2 h at 37°C in Tris–HCl 50 mM, pH 7.4. After incubation, the samples were centrifugated (2,000 rpm, 5 min) and the supernatant was analysed by MALDI-TOF/MS as follows. Aliquots (1 μL) were added to 1 μL of alpha-ciano-4-hydroxycinnamic acid (10 mg/mL) matrix solution, spotted onto a stainless steel MALDI target plate and dried at room temperature before analysis. Mass spectra were obtained with a Bruker Daltonics Microflex LT instrument operating in linear, positive ion mode previously calibrated with angiotensin I, angiotensin II, somatostatin and bradykinin. For the analysis of peptide release, mass spectra were acquired using the following instrument parameters: pulsed ion extraction delay of 30 ns, ion source voltage one, 20 kV, ion source voltage two, 18.65 kV, and ion source lens voltage 7.1 kV. For each sample, mass spectra were acquired by accumulating 50 laser shots at 50% laser power in the m/z range of 800–2600 Da.

### SDS-PAGE analysis of biotin-HK hydrolysis by falcipain-2 and falcipain-3

Human single chain HK was biotinylated (Biotin-HK) as previously described [31]. Biotin-HK(0.4 μM) was incubated with falcipain-2 (0.2 μM) and falcipain-3 (0.2 μM) in sodium acetate buffer 100 mM, pH 5.5, for 1 h at 37°C and the reactions were stopped by boiling. Samples containing only biotin-HK, falcipain-2 or falcipain-3 were also analysed as experimental controls, and were assayed in the same conditions. The samples were submitted to western blotting and nitrocellulose transblots were washed with PBS and incubated with PBS / Tween 0.01% / BSA 1% solution. After extensively washes with PBS, the membrane was incubated with streptavidin–peroxidase conjugated (1:1000) for 1 h at 4°C and the results were obtained by degradation of peroxidase substrate 4-Chloro-1-naphthol (SIGMA) (30 mg 4-Chloro-1-naphthol solution in 10 mL of methanol to 50 mL of PBS) at room temperature.

### Guinea pig ileum contraction induced by kinin peptide generation

The biological activity of the released kinins was measured as isotonic contraction on isolated guinea pig terminal ileum. The isolated organ was suspended in a 5 mL bath in Tyrode’s solution (135 mM NaCl, 2.7 mM KCl, 1.36 mM CaCl_2_, 0.5 mM MgCl_2_, 0.36 mM NaH_2_PO_4_, 11.9 mM NaHCO_3_, 5.04 mM glucose) at 37°C, pH 7.4. The sensitivity of the response was calibrated with standard solutions of bradykinin (1–10 nM). HK (0.33 μM) was incubated with isolated *P. chabaudi* trophozoites (10^4^ cells) and with recombinant falcipain-2 (46 nM) or falcipain-3 (18 nM). After 10 min, parasites, when present, were pelleted by centrifugation, and supernatants were added to the bath and the isotonic contraction recorded as described previously [20]. The volume of supernatant was adjusted so that the released kinin fit inside the dose–response curve for bradykinin. Similar experiments were performed in the presence of 20 nM of the bradykinin B2 receptor antagonist HOE-140 (D-Arg-Arg-Pro-Hyp-Gly-b-[2-thienyl]Ala-Ser-D-tetrahydroisoquiniline-3-carboxylicacid-octahydroindole-2-carboxylic acid-Arg), which was pre-incubated for 2 min. Falcipain-2 (46 nM) and falcipain-3 (18 nM) were pre-activated with 2 mM DTT for 5 minutes and then incubated with HK (0.48 μM) in sodium acetate buffer 0.1 M, pH 5.5 at 37°C for six hours. The reaction was then kept in ice. Afterwards, 100μL of the solution was added to the system containing the guinea pig ileum in Tyrode’s solution. As a control, falcipains were also assayed in the same system after 10 min incubation with the specific cysteine protease inhibitor E-64 (10 μM).

### Confocal Microscopy of FITC labelled HK

FITC (Sigma) was conjugated to HK according to manufacturer’s specifications. To investigate the permeability of infected erythrocytes to HK, *Plasmodium chabaudi* and *Plasmodium falciparum* infected cells were ressuspended in PBS and 10^4^ cells were placed on microscopy plates pre-incubated with poly-L-lysine (1 h at room temperature) to enhance cell adhesion. Afterwards, labelled HK (1 μg) was added to the plates and incubated for 1 h at 37°C. Fluorescence images were obtained with a confocal microscope (Carl Zeiss LSM-510 META) with excitation wavelength of 488 nm and emission of 505–550 nm.

### Intracellular calcium measurement in HUVEC cells stimulated with kinin peptides

Human umbilical vein endothelial cells were cultured in RPMI 1640 (GIBCO) containing 10% heat-inactivated fetal bovine serum (FBS), 24 mM sodium bicarbonate, 40 mg/mL gentamicin, 10 mM HEPES, pH 7.4 at 37°C in a 5% CO_2_ humidified atmosphere. 5 × 10^4^ cells were plated on 60 mm dishes (Costar 3260), and incubated in culture medium containing 5 μM Fluo-3 AM (Invitrogen Corporation, CA) for 1 h at 37°C, followed by 3 washes with culture medium to discard remaining extracellular probe. The cells were then placed into a culture chamber at 37°C on the stage of a confocal microscope (Zeiss LSM 510 META) with excitation at 488 nm and emission at 505–530 nm. After calcium probe incubation, assays were performed with the antagonists Des-Arg^9^[Leu^8^]-BK (4 μM) and HOE-140 (10 μM). The antagonists were incubated for 10 min before the addition of: 20 μg kininogen (HK), 10 µL of culture supernatant after culturing 10^4^*P. falciparum* for 30 minutes without HK (Pf - HK); 10 μL of culture supernatant after culturing 10^4^*P. falciparum* for 30 minutes with HK (1.9 μg/μL) (Pf + HK); or 5 μM THG. Fluorescence data were normalized as F/Fmax, where Fmax represents maximal intracellular fluorescence obtained with Ca^2+^ released from ER with addition of THG (Ca^2+^ATPase inhibitor).

## Results

### Kinin peptides are generated by *Plasmodium* parasites

**Figure 1:**
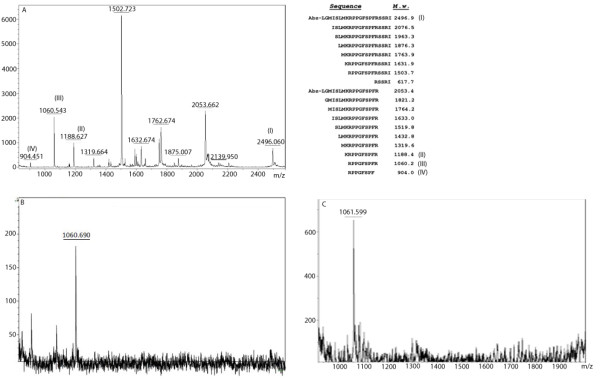
**Mass spectrometry spectrum of extracellular medium of*Plasmodium falciparum*or*Plasmodium chabaudi*parasites.(A)** Isolated *Plasmodium chabaudi* parasites at the trophozoite stage (10^4^ cells) were incubated with 30 μM of Abz–MISLMKRPPGFSPFRSSRI-NH2 (I) for 10 min, at 37°C and peptides generated are described in the right panel. Kinin peptides detected in (A) are: Lys-BK (II), BK (III) and des-Arg^9^-BK (IV). **(B)** Detection of BK (bradykinin) peptide in extracellular medium after incubation (1 h) of *Plasmodium chabaudi* with 0.35 μM kininogen (HK). **(C)** BK detection after incubation (1 h) of *Plasmodium falciparum* (10^4^ cells) with HK (0.35 μM).

In order to investigate the capability of the human and rodent malaria parasites to generate kinins, *Plasmodium falciparum* and *Plasmodium chabaudi* isolated trophozoites were incubated with a synthetic fluorogenic kininogen fragment, which contains the bradykinin sequence (Figure 1). MALDI-TOF analysis of the extracellular medium of cell incubations showed cleavage of this synthetic peptide generating fragments characterized as Lys-BK (KRPPGFSPFR), BK (RPPGFSPFR) and des-Arg^9^-BK (RPPGFSPF), according to their calculated molecular mass (Figure 1A). Next, the parasites were incubated with the plasma BK precursor HK, to assess the generation of kinins from the physiological source. After 1 hour of incubation, BK was detected in both assays (Figure 1B and 1C).

**Figure 2:**
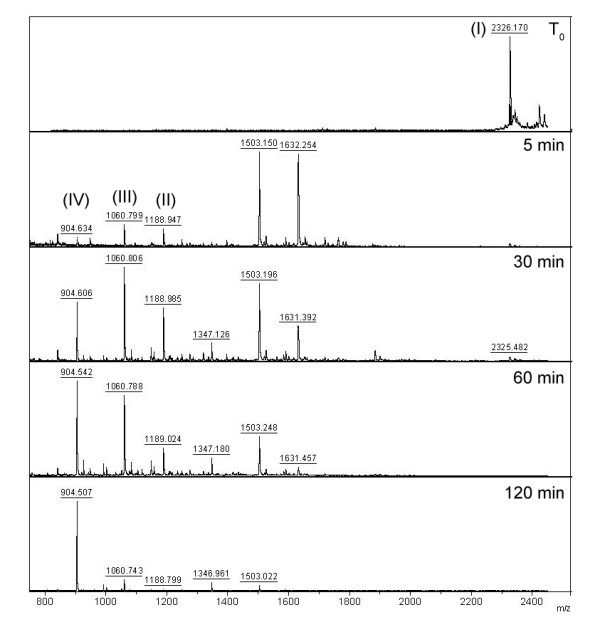
**Mass spectrometry analysis of the time course hydrolysis of BK-related peptides by*Plasmodium falciparum*parasites.** The substrate Abz–MISLMKRPPGFSPFRSSRI-NH_2_ (I) (30 μM) was incubated with isolated *P. falciparum* parasites (10^4^ cells) for the indicated times (0-120 min) and the supernatant was analysed by MALDI-TOF/MS, detecting the kinin peptides: Lys-BK (II), BK (III), and des-Arg^9^-BK (IV).

Thereafter, a time course analysis of the processing of the fluorogenic kininogen fragment and of HK by *Plasmodium falciparum* was performed (Figure 2). The synthetic peptide was incubated with parasites, and the extracellular medium was scrutinized by mass spectrometry over two hours and presented as relative kinin percentage (Table 1). The results showed time-dependent cleavage of the substrate, generating within 5 minutes two major fragments of 1503 and 1663 Da and peptides with molecular mass corresponding to Lys-BK, BK and des-Arg^9^-BK. The amount of kinins generated after 30 minutes were higher, indicating continuous processing of the substrate by the parasites. After one hour, the 1060 and 904 Da peaks, corresponding to BK and des-Arg^9^-BK, were still visible, but the larger fragments were entirely degraded. After two hours, the only kinin peptide detected was des-Arg^9^-BK.

**Table 1:** **Mass spectrometry analysis of the BK-related peptide by*Plasmodium falciparum*parasites**

	Kinins (%)		
Time (minutes)	des-Arg^9^-BK (IV)	BK (III)	Lys-BK (II)
0	0	0	0
5	15	48	37
30	28	43	29
60	48	39	13
120	89	10	0

Relative percentage (%) of each kinin generated after incubation of *Plasmodium falciparum* parasites with Abz–MISLMKRPPGFSPFRSSRI-NH2 as presented in Figure 2

### *Plasmodium* parasites take up HK

**Figure 3:**
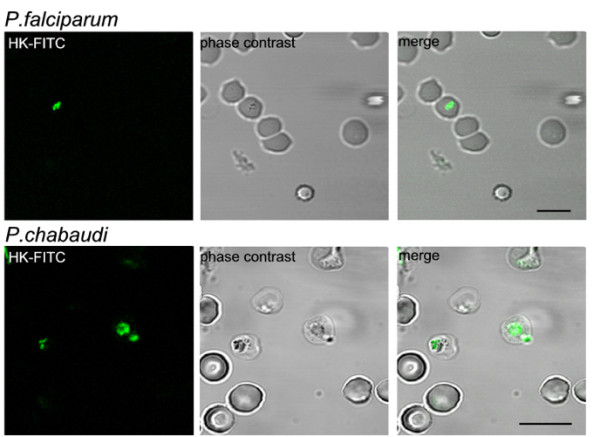
**Intracellular uptake of HK in iRBC.** Erythrocytes infected with *Plasmodium falciparum* and *Plasmodium chabaudi* plated in microscopy dishes were incubated with HK-FITC protein (1 μg) for 1 h at 37°C, and images performed by fluorescence confocal microscopy *Scale bar 10 μm.

Malaria parasites might degrade HK after intracellular uptake or by means of an exported protease. To determine whether the peptide is imported, HK was labeled with FITC and infected erythrocytes were evaluated by confocal microscopy. The resultant images clearly show fluorescent HK co-localized within the parasites, indicating the uptake of HK by *Plasmodium falciparum* and *Plasmodium chabaudi* (Figure 3). Uninfected erythrocytes did not show fluorescence.

### Falcipains mediate HK cleavage

**Figure 4:**
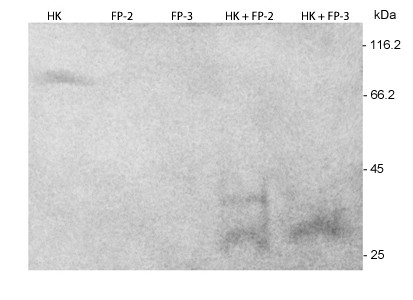
**SDS-PAGE analysis of biotin-HK hydrolysis by falcipain-2 and falcipain-3.** Biotin-HK (0.4 μM) was incubated with each falcipain protease (0.2 μM) for 1 h, and the individual components (HK, FP-2, FP-3) and combined reactions were then separated by SDS-PAGE and their products visualized by nitrocellulose transblots staining with streptavidin–peroxidase conjugated (1:1000/1 h) and its substrate 4-Chloro-1-naphthol.

To study the involvement of *Plasmodium* cysteine proteases in kininogen hydrolysis, recombinant falcipain-2 and falcipain-3 were incubated with HK for 1 h and cleavage products were analysed by SDS-PAGE and immunoblotting. Both proteases cleaved HK, but they produced different cleavage products (Figure 4).

### Fragments generated by falcipains and *Plasmodium chabaudi* possess biological activity in guinea pig ileum

**Figure 5:**
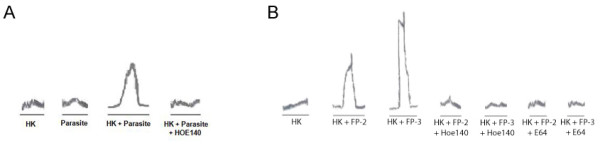
**Effect of generated kinins by Plasmodium intracellular hydrolysis on ileum contraction.** (A) Guinea pig ileum isotonic contraction measurements induced by kinin generated from HK by *Plasmodium chabaudi* parasites. Traces of ileal contraction are shown after 5 min. incubations with HK; *Plasmodium chabaudi* parasites; HK (0.33 μM) and parasites; and HK, parasites, and HOE-140 (20nM). (B) Guinea pig ileum contraction induced by kinin generated by falcipain-2 and 3 hydrolysis from HK. Traces of ileal contraction are shown after 5 min. incubations as labeled, including 0.48 μM HK, 46 nM falcipain-2 (FP-2), 18 nM falcipain-3 (FP-3), and/or 10 μM E-64.

To assess the bioactivity of HK-derived peptides generated by *P. chabaudi*, falcipain-2 and falcipain-3, their ability to induce ileum contraction, which is mediated by activation of B1 and B2 receptors [13] was studied. Neither HK nor *P. chabaudi* supernatant stimulated ileum contraction (Figure 5A). In contrast, extracellular medium of parasites incubated with HK promoted contraction of the muscle. Contraction was abolished when HOE-140, a B2 receptor antagonist, was added to the medium, showing participation of the B2 receptor. The same approach was used to test the activity of HK fragments resulting from falcipain cleavage. HK processed by falcipain-2 and falcipain-3 promoted ileum contraction, which was prevented by HOE-140 (Figure 5B). When HK was incubated with falcipains in the presence of E-64, a specific cysteine protease inhibitor, the contraction response was also ablated, showing that the proteolytic activity of the enzymes was required for the production of active peptides.

### Calcium signalling by kinin peptides generated by *Plasmodium falciparum*

**Figure 6:**
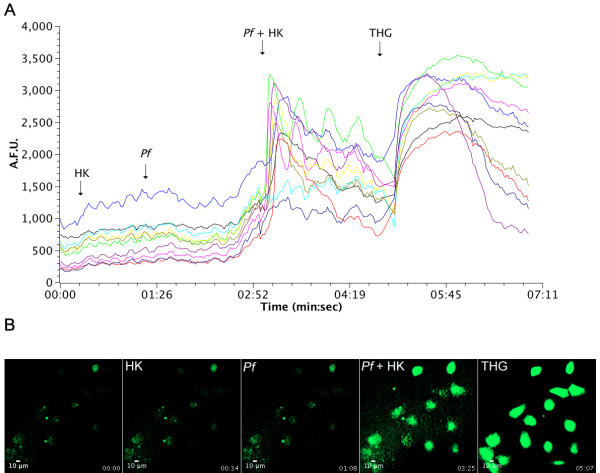
**Effect of generated kinins peptides by*Plasmodium*on HUVEC intracellular Ca**^**2+**^**mobilization.** Malaria parasites (10^4^ cells) were incubated with 20 μg of kininogen for 30 minutes, and extracellular medium was then added to HUVECs loaded with the Fluo-3 AM fluorescent calcium probe and measured in confocal microscopy. (A) Intracellular calcium fluorescence of cells incubated with HK (20 μg), 10 μl of parasite culture medium without HK (Pf), 10 μl of parasite culture medium after incubation with 1.9 μg/μL of HK for 30 min (Pf + HK) and thapsigargin (THG, 5 μM), a Ca^2+^-ATPase inhibitor. Each line represents results for a single cell. (B) Time lapse calcium fluorescence images of HUVECs, with the times of addition of components indicated.

To further assess the biological activity of HK-derived peptides exported from infected erythrocytes, the effects of these peptides on the intracellular calcium mobilization of human umbilical vein endothelial cells (HUVECs) were measured. When HK alone or *Plasmodium falciparum* culture supernatants alone were added to the cells, no change was detected in cell fluorescence. However, when the extracellular medium from *Plasmodium falciparum* incubated with HK was added, a clear increase in [Ca^2+^]_i_ was observed (Figure 6). When the parasites were treated with 5 μM E-64 prior to the addition of HK, the generated fragments were unable to elicit a calcium response.

**Figure 7:**
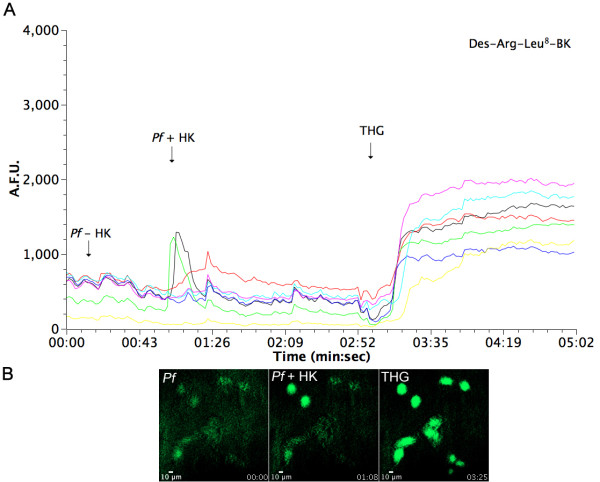
**Effect of generated kinins on HUVECs intracellular Ca**^**2+**^**mobilization in the presence of the B1 receptor antagonist Des-Arg**^**9**^**Leu**^**8**^**-BK.** Malaria parasites (10^4^ cells) were incubated with 20 μg of HK for 30 minutes, and extracellular medium was then added to HUVECs loaded with the Fluo-3 AM fluorescent calcium probe and previously incubated for 10 min with Des-Arg^9^Leu^8^-BK (4 μM). (A) Intracellular calcium fluorescence of cells incubated with HK (20 μg), 10 μl of parasite culture medium without HK incubation (Pf), 10 μl of parasite culture medium after incubation with 1.9 μg/μL of HK for 30 min (Pf + HK) and thapsigargin (THG 5 μM), a ER Ca^2+^-ATPase inhibitor. Each line represents results for a single cell; (B) Time lapse calcium fluorescence images of HUVECs measured by confocal microscopy, with the times of addition of components indicated.

**Figure 8:**
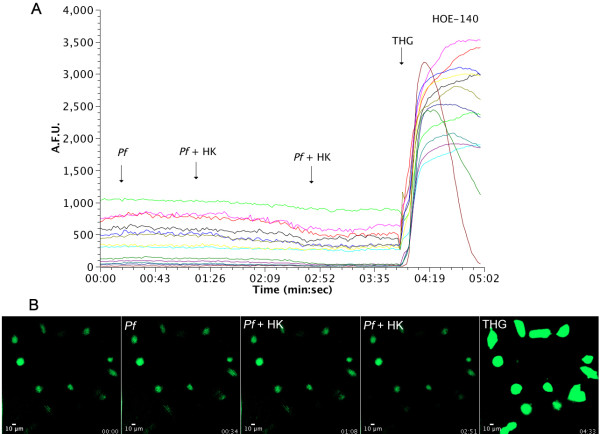
**Effect of generated kinins on HUVECs intracellular Ca**^**2+**^**in the presence of the B2 receptor antagonist HOE-140.** Malaria parasites (10^4^ cells) were incubated with 20 μg of HK for 30 minutes, and extracellular medium was then added in HUVECs loaded with the Fluo-3 AM fluorescent calcium probe and incubated previously for 10 min. with HOE-140 (10 μM). (**A**) Intracellular calcium fluorescence of cell incubated with HK (20 μg), 10 μl of parasite culture medium without HK incubation (Pf), 10 μl of parasite culture medium after incubation with 1.9 μg/μL of HK for 30 min. (Pf + HK) and thapsigargin (THG 5 μM), a ER Ca^2+^-ATPase inhibitor. Each line represents results for a single cell; (**B**) Time lapse calcium fluorescence images of HUVECs, with the times of addition of components indicated.

**Figure 9:**
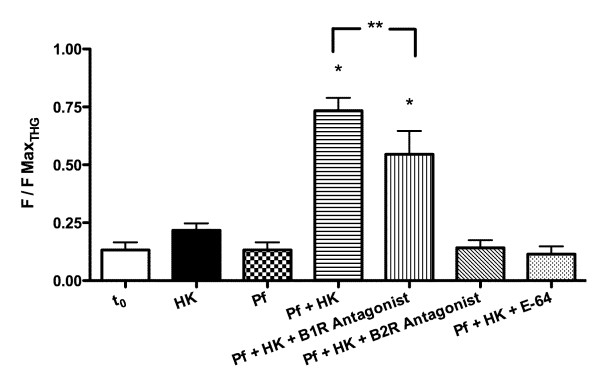
**Intracellular calcium mobilization in HUVECs cells after B1 and B2 receptors activation by kinin peptides.** HUVECs were loaded with Fluo-3 AM and incubated with HK (20 μg) (HK); 10 μl of parasite culture supernatant without HK (Pf); 10 μl of parasite culture supernatant after incubation with 1.9 μg/μL of HK for 30 min. (Pf + HK); 10 μl of parasite culture supernatant after incubation with 1.9 μg/μL of HK for 30 min and pre-incubation (10 min.) with 4 μM Des-Arg^9^Leu^8^-BK (Pf + HK + B1 antagonist); or 10 μM HOE-140 (Pf + HK + B2 antagonist); 10 μl of parasite culture supernatant after incubation with 1.9 μg/μL of HK for 30 min (Pf + HK) pre-incubated with 5 μM E-64 (Pf + HK + E64). Data were normalized as the ratio between measured fluorescence (F) and maximal fluorescence (FmaxTHG) obtained with addition of 5 μM THG. *Denotes statistical significance with respect to all groups values p < 0.01 (n = 3; 30 cells); **Statistical significance of p < 0.05 between Pf + HK and Pf + HK + B2 antagonist (n = 3; 30 cells) in One-way ANOVA test and Newman-Keuls Multiple Comparison test.

To evaluate receptor activation in response to peptides generated by parasite proteolysis of HK, HUVECs were pre-incubated with the B1 receptor antagonist Des-Arg^9^[Leu^8^]-BK and the B2 receptor antagonist HOE-140 before stimulus and then measured intracellular fluorescence (Figure 7). [Ca^2+^]_i_ was partially inhibited in the presence of Des-Arg^9^[Leu^8^]-BK when compared to control conditions without antagonist. In contrast, pre-incubation with HOE-140 led to complete inhibition of intracellular calcium release (Figure 8). These results, summarized in Figure 9, show that peptides derived from HK processing by malaria parasites are biologically active, and transduce their signal mainly by activation of B2 receptors in HUVEC cells.

## Discussion

The results reported here show that *Plasmodium* can process the plasma protein kininogen and generate peptides that may modulate hemodynamics in the host. *P. falciparum* and *P. chabaudi* are able to internalize and process kininogen, with subsequent release of kinins (des-Arg^9^-BK, Lys-BK and BK). This processing is inhibited by the cysteine protease inhibitor E-64 and reproduced *in vitro* by isolated falcipain-2 and falcipain-3, suggesting roles for these proteases in kininogen processing. Kininogen peptides were biologically active, as demonstrated by their ability to induce ileum contraction and activate endothelial cells via calcium signaling, both principally through B2 receptor activation.

The processing of plasma macromolecules by the malaria parasites has already been reported [32-35]. However, parasites have not previously been shown to modulate host physiology by these events. Kininogen is a multipurpose plasma protein that plays essential roles in modifying vascular haemodynamics, controlling vascular tone and maintaining blood pressure [16]. The results here reported suggest that parasites can metabolize peptides and proteins from plasma or from the endothelial cell surface, releasing active peptides into the extracellular milieu and thereby impacting upon host haemodynamics.

The processing of a plasma protein by falcipains *in vitro* suggests a second physiologic function for these proteases in addition to the hydrolysis of haemoglobin [7]. Interestingly, falcipain-2 and falcipain-3 generated distinct proteolytic profiles, suggesting that the activities were not redundant. However, the participation of other proteases in this process cannot be excluded. Recently, the *Plamodium falciparum* aminopeptidase PfAPP was shown to localize to the food vacuole and cytosol of the parasite [34], suggesting potential roles in the hydrolysis of both haemoglobin and HK. Interestingly, recombinant PfAPP was able to hydrolyse bradykinin [34]. The mass spectrum data showed the removal of amino acid residues from the N-termini of proteins, supporting the involvement of aminopeptidases in kinin processing.

It was of interest to determine if peptides generated by parasites and isolated falcipains possess biological activity and exert a specific physiologic effect. Alternatively, they might be generated simply for acquisition of nutrients, as appears to be the case for haemoglobin. Indeed, generated peptides promoted guinea pig ileum contraction, which was abolished in the presence of the B2 receptor antagonist HOE-140. Further, the peptides stimulated Ca^2+^ release in endothelial cells and this process was also blocked by HOE-140. Thus, proteolysis of kininogen by malaria parasites generates biologically active peptides. This effect may explain clinical features of malaria, which is commonly accompanied by alterations in vascular haemodynamics.

Another potential role for BK and B2 receptor stimulation is attenuation of host systemic damage during infection by anti-apoptotic or other protective effects. Similar protective effects were observed in cardiac, renal and liver damage models [36]. For instance, B2 activation induces a hypertensive hepatic response [37], which could play a role in malaria liver stage physiology, with modulation of hepatic blood flow and parasite distribution in the tissue.

In the late 60s, some studies suggested that kinins could be involved in the pathological manifestations of malaria [27-29]. However, these works were not conclusive and, despite the evidences, no more reports correlating kinins with malaria could be found in literature. This new set of evidences points out to the participation of the kinins in the pathophysiology on *Plasmodium* infection. This process appears to depend on endothelial cell activation, which may be promoted by proinflammatory agents such as cytokines (e.g. Tumor Necrosis Factor), chemokines and infected erythrocytes [33,35,38]. This activation leads to enhanced expression of genes that are involved in inflammation and apoptosis [39]. Nitric oxide production is induced by kinin receptors, has been shown to mitigate brain haemorrhages and vascular damage in a murine cerebral malaria model [40] and may modulate host haemodynamics during malarial infection. The modulation of vessel caliber by vasoactive peptides might be important as a mechanism to prevent thrombosis by the enhanced cytoadherence and promote better blood flow in the microcirculation, which is impaired by the malarial infection, in a process that confers some protection to the host, and consequently ensuring that the pathogen can continues its life cycle.

## Conclusions

**Figure 10:**
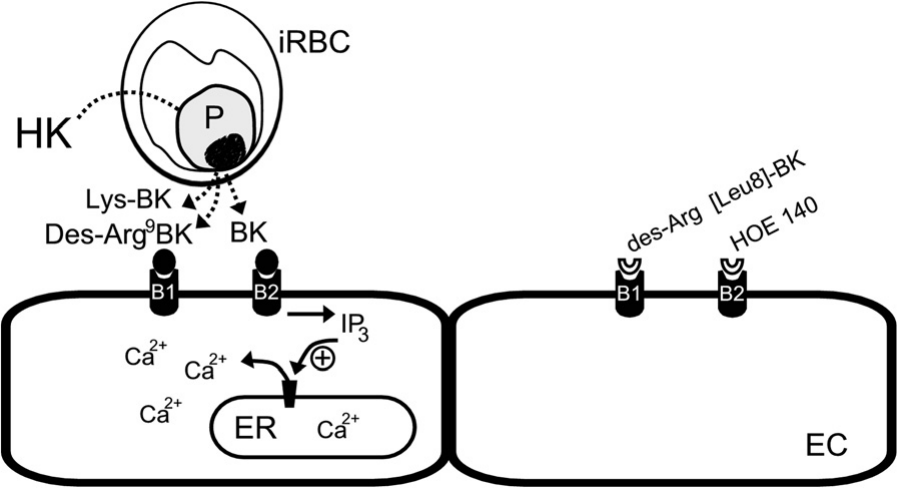
**Schematic model of endothelial cell activation by kinin peptides generated by*Plasmodium*infected erythrocytes.** iRBC possess permeability and transport pathways involved in plasma protein and peptide acquisition. Kinin peptides detected: bradykinin (BK), Des-Arg^9^-BK, Lys-BK; antagonists used for B1 (Des-Arg^9^[Leu^8^]-BK) and B2 (HOE-140) receptors; iRBC – infected erythrocytes; ER- endoplasmic reticulum; HK – human kininogen; EC – endothelial cell.

Taken together, the results reported point to a potential role for the hydrolysis of HK by malaria parasites in the pathophysiology of malaria. The hydrolysis of plasma proteins suggests an intricate relationship between host and pathogen, and may represent a key adaptation by malaria parasites to facilitate host infection. A schematic model of endothelial cell activation by kinin peptides generated by *Plasmodium* is shown in Figure 10. The malaria parasite may alter the concentration of systemic or local kinins to induce vasodilatation and endothelial permeability, which may facilitate parasite survival. However, further studies are necessary to evaluate the biological importance of kinin production during plasmodial infection.

## Misc

Piero Bagnaresi, Nilana MT de Barros, contributed equally to this work

## Competing interests

The authors declare that they have no competing interest.

## Authors’ contributions

PB carried out the confocal microscopy experiments, participated on the design of the study and drafted the manuscript. NMTB carried out the ileum contractions assays, participated on the design of the study and drafted the manuscript. DMA carried out the spectrometry analysis. PMSM participated in spectrometry assays and microscopy. RPG, JBP, MAJ, LJ and PJR participated in the design of the study. AKC and MLG conceived and coordinated the study, and helped to draft the manuscript. All authors read and approved the final manuscript.
